# Decentralising TB services for children: quality of care is fundamental

**DOI:** 10.5588/ijtldopen.24.0652

**Published:** 2025-05-12

**Authors:** S.M. Graham, S. Zawedde-Muyanja, M. Palmer, R.A. Dlodlo

**Affiliations:** ^1^ International Union Against Tuberculosis and Lung Disease, Paris, France;; ^2^ The University of Melbourne Department of Paediatrics, Royal Children’s Hospital, Melbourne, VIC, Australia;; ^3^ The Infectious Diseases Institute, College of Health Sciences, Makerere University, Kampala, Uganda;; ^4^ Desmond Tutu TB Centre, Department of Paediatrics and Child Health, Stellenbosch University, Cape Town, South Africa.

**Keywords:** tuberculosis, TPT, paediatric TB, chest X-ray, CXR, decentralisation

As reported by the WHO, current gaps in the detection, treatment and prevention of TB are widest in children, with case detection or ‘treatment coverage’ being lowest in those less than five years.^1^ Coverage of TB preventive treatment (TPT) in eligible child contacts is also far below global targets, despite long-standing compelling evidence that young children are at high risk for severe disease following infection and that TPT is highly effective in reducing that risk.^2,3^ Closing these detection and prevention gaps will reduce global child morbidity and mortality. Expertise and services for children with TB disease or infection are still largely centralised to tertiary care facilities in many settings. Diagnosing and treating children in primary and secondary care could improve access and avoid unnecessary referrals.^4^ Specialist paediatric centres are also often not well connected to healthcare services for TB in adults, with consequently missed opportunities for prevention in children. Community-based contact investigation could potentially increase early treatment of TB in all ages and TPT uptake in eligible contacts, improve outcomes and reduce unnecessary costs for both families and the healthcare system.

Since 2006, the WHO has recommended a symptom-based approach to contact management and TPT for young child contacts for implementation at the community level.^5^ The recent WHO consolidated guidelines recommend that ‘decentralised tuberculosis services may be used in children and adolescents with signs and symptoms of tuberculosis and/or in those exposed to tuberculosis’.^6^ It was observed that strengthening services at primary or secondary care facilities can increase case detection, improve treatment outcomes and introduce contact investigation and TPT coverage.^4,7^ A recent cluster randomised-controlled trial found that community-based services in Uganda and Cameroon achieved significant improvements in contact screening coverage, as well as TPT initiation and completion, compared to facility-based services.^8^ Furthermore, this intervention was cost-effective with reduced costs to families.^9^ Although a decentralised model can improve access to care, it is critical to maintain the quality of care at all levels.^10^ Healthcare workers (HCWs) at decentralised facilities often lack confidence or knowledge on how to manage TB in children. A study in Kenya reported increased TB detection at outpatient facilities and improved contact screening and TPT uptake by providing financial support for travel costs to the clinic. However, it was noted that screening for presumptive TB and diagnostic pathways needed optimisation.^11^ Opportunities for bacteriological confirmation have improved with increased access to molecular rapid diagnostics, which often simultaneously test for drug resistance, and a wider array of non-invasive sampling techniques for those who cannot provide sputum.^6^ However, most children with TB continue to be diagnosed clinically due in part to unique factors that impact the performance of existing diagnostics. HCWs need support to develop expertise in clinical diagnosis and to apply it within the context of striving to provide quality care for all sick children. Increasing attention to a specific disease, such as TB in children, can cause a swing from under- to over-diagnosis. Whilst some over-diagnosis with treatment is accepted and preferable to under-diagnosis, the consequences of unnecessary treatment for the child, their caregivers and the resource-limited health services are not trivial.^10^

Structured approaches to support clinical diagnosis in children have been developed over many years.^12^ They commonly include weighted scoring systems that consider age, clinical features, nutritional assessment, TB exposure history, evidence of infection and radiological findings. There is, however, wide variability of yield for case detection with poor agreement between approaches.^12^ Specific structured approaches have recently been developed and validated for children living with HIV or with severe acute malnutrition.^13,14^ Treatment decision approaches should also accommodate practical management issues such as evaluating any sick child for severity of illness, comorbidities, urgency of treatment and indications for referral.^15^ Additionally, a decision to treat for TB does not need to be taken in a single consultation and follow-up visits can be informative.

The Union has developed a job aide that supports HCWs in providing care, including at the primary care level, using a structured management approach that has been updated to include new diagnostics.^16^ It starts with triage for urgent care and ends with the option of follow-up for children with presumptive TB who are not severely ill and in whom clinical diagnosis remains uncertain. The WHO operational handbook includes two scoring options for children without bacteriological confirmation – one when CXR is available and one when not.^17^ These scores were derived using data from >4,000 children with the cut-off deliberately selected to prioritise sensitivity (limit under-diagnosis) over specificity (accept over-diagnosis).^18^ The scores are nested within a treatment decision approach, which is now being evaluated for effectiveness and feasibility in multiple countries. The inclusion of CXR in treatment decision approaches aligns with standard practice to support the diagnosis of intrathoracic TB in children.^10,15,16^ Aside from limitations of access, a major challenge for decentralisation is the capability of HCWs to interpret CXRs; specifically, the capability to determine if a CXR is of sufficient quality for interpretation, if abnormal or normal, and whether the abnormalities are consistent with TB. In 2022, The Union’s ‘Diagnostic CXR atlas for tuberculosis in children’ was substantially updated to support interpretation and include the classification of severe versus non-severe intrathoracic TB, which informs treatment duration.^19^ The atlas is complimented by a CXR Image Library and an on-line course that are freely accessible.^19,20^

CXR is increasingly accessible using mobile platforms. The development of artificial intelligence software, though not yet validated in children, offers the potential of standardised CXR interpretation with accuracy approximating human experts that could be made widely available. CXR is recommended in addition to symptom screen for active case-finding in adults, including of asymptomatic TB, as well as to exclude active TB in child contacts of 5 years and older to determine eligibility for TPT.^5,17^ The prevalence of asymptomatic TB in children in the community is not known. Studies of children recently exposed to *Mycobacterium tuberculosis* report it to be uncommon.^21^ There is strong evidence and programmatic experience that young child contacts can safely and effectively be initiated on TPT based on being asymptomatic and not malnourished.^17,22^ Community case-finding studies using mobile CXR are ongoing that are evaluating the age-related prevalence of asymptomatic TB in children of 5 years and older.

Effective decentralisation that attends to quality person-centred care requires HCWs at peripheral levels of care to be empowered with clinical confidence and expertise supported by ongoing mentorship, using local data for continuous quality improvement.^7^ High rates of treatment success remain critical, and the availability of drugs, including child-friendly options, must be ensured. Decentralisation will likely lead to an increased caseload and workload. However, this can be managed without compromising (potentially even improving) outcomes as decentralisation potentially facilitates retention in care and treatment adherence.^7^ The WHO recommendation on decentralised models of care acknowledges the need for high-quality evidence of benefits, impact on treatment outcomes, health system resource requirements, and cost-effectiveness in various settings. Person-centred care has always been fundamental to care for sick children –individual considerations of age, development, weight, cognitive ability, risk and vulnerability of the child in addition to the characteristics and needs of their caregivers.^15^ Indeed, this may be why children have often been relegated to the ‘too-hard basket’ for TB. However, in contrast to tertiary facilities, care for children and adults at the peripheral level is usually provided by the same healthcare worker. Although the DETECT project focussed on supporting HCWs to improve diagnosis in children, adult case detection improved by 30%.^7^ Strengthening health systems for children with TB is a priority for resource-limited settings.^22^ Decentralisation of services can improve access to care in children and adults, and resources are increasingly available to support policy translation to optimise care at all levels. Resources such as The Union’s CXR Atlas^19^ provide on-line training for individuals or groups without the need for expensive face-to-face workshops and with flexible scheduling. In 2023–2024, WHO developed two short e-learning courses for child and adolescent TB.^23,24^ One aims to inform HCWs on how to diagnose, treat and prevent TB, while the other aims to support programmatic implementers and trainers, including stressing procurement needs of diagnostics and child-friendly treatments.

The Union has developed a comprehensive course with materials for facilitators that cover a full range of topics and multiple interactive opportunities for participants using adult learning principles. The materials were piloted in Lesotho in 2024 with participants from 11 African countries representing national child and adolescent TB working group members. The curriculum was refined to incorporate feedback, and the final version was recently launched.^25^ The training includes the active use of routinely available TB data at all levels of health services as monitoring age-disaggregated and facility-level key indicators provide a basis for site-specific support and actionable feedback. Data are fundamental to improving the quality of services, and there is enormous potential and scope for operational research. Evaluation of the new Union hybrid training materials as they are cascaded by countries to all levels of care will be important in terms of uptake and acceptability, but most importantly, to evaluate the impact on coverage and quality of care.
